# Synergistic Gypsum–Carbonation Strategy and Non-Contact ITZ Quantification for CFBFA Artificial Aggregate Concrete

**DOI:** 10.3390/ma18225240

**Published:** 2025-11-19

**Authors:** Nuo Xu, Mingyi Guo, Yiheng Chen, Rentuoya Sa, Mao Huo, Suxia Ma

**Affiliations:** 1 Shanxi Province Key Laboratory of High Efficient & Clean Combustion and Utilization of Circulating Fluidized Bed, Taiyuan University of Technology, 79 Yingze West Street, Taiyuan 030024, China; 2 School of Electric Power Engineering, Shanxi College of Technology, 11 Changning Street, Shuozhou 036000, China

**Keywords:** circulating fluidized bed fly ash, Interfacial transition zone, carbonation curing, gypsum activation, double-peak gradient method, microstructure–mechanical property relationship

## Abstract

This study explores an integrated strategy combining gypsum activation and pressurized flue gas heat curing (FHC) to enhance the interfacial transition zone (ITZ) in concrete incorporating over 80% circulating fluidized bed fly ash (CFBFA)-based artificial coarse aggregates. The inherently weak ITZ, characterized by low bonding strength and high porosity, remains a major limitation to the mechanical performance of CFBFA-based concrete. Gypsum promotes the formation of ettringite (AFt) and facilitates the development of a dense CaCO_3_ shell through enhanced carbonation. Their synergistic effect improves microstructural homogeneity and reduces crack connectivity at the interface. A novel grayscale image-based double-peak gradient method is developed for non-contact, quantitative measurement of ITZ thickness, revealing a strong inverse correlation (R^2^ = 0.87) between ITZ thickness and compressive strength. Microstructural analyses confirm that the dual treatment significantly refines the ITZ, resulting in denser aggregate interiors, improved matrix continuity, and more structurally integrated interfaces. The failure mode correspondingly shifts from interface-dominated fracture to composite-controlled behavior. These findings demonstrate the effectiveness of the FHC–gypsum approach in tailoring ITZ morphology and enhancing mechanical integrity, offering a viable pathway for high-performance, low-carbon cementitious composites utilizing industrial by-products.

## 1. Introduction

In recent decades, the construction industry has faced increasing pressure to adopt sustainable and low-carbon practices. In response to global carbon reduction targets, urgent action is needed to limit CO_2_ emissions. Traditional concrete production, which relies heavily on natural aggregates, has significantly contributed to resource depletion and environmental degradation [1]. This has led to the development of artificial aggregates (AAs) derived from industrial by-products as a key strategy for reducing the ecological footprint of cement-based materials [2,3]. Among these, circulating fluidized bed fly ash (CFBFA), a by-product of coal combustion in CFB boilers, has garnered considerable attention due to its abundant availability and potential for sustainable utilization [4]. However, the comprehensive utilization rate of CFBFA remains notably low, with less than 25% of the total output being recycled [5,6,7]. These inherent chemical characteristics make it suitable for producing artificial aggregates when combined with chemical activators and appropriate curing strategies. Circulating Fluidized Bed Fly Ash (CFBFA) contains Al_2_O_3_, SiO_2_, CaO, SO_3_, Fe_2_O_3_ and MgO, with pozzolanic activity and self-hardening properties [8,9,10]. To enhance SO_2_ removal efficiency with sulfur-fixing agents like limestone or dolomite, its Ca/S molar ratio is typically increased to 2.0–2.5, leading to significant f-CaO and desulfurization product CaSO_4_, making it suitable for use in cement production [11,12], road base materials, concrete, and artificial aggregates [13]. Given that aggregates account for up to 75% of concrete by volume, large-scale utilization of CFBFA for artificial aggregate production holds significant promise. However, the use of CFBFA as a precursor for artificial coarse aggregates faces challenges, particularly its low hydraulic reactivity and the formation of a weak interfacial transition zone (ITZ) with the surrounding cement matrix, which compromises the overall mechanical properties and durability of concrete.

The interfacial transition zone (ITZ) is a critical weak point in concrete, especially in recycled or artificial aggregates like those from circulating fluidized bed fly ash (CFBFA) [14,15]. Yue et al. [16] highlighted that in recycled aggregate concrete (RAC), the ITZ weakens under chloride exposure, reducing concrete properties. Similarly, Kirthika and Singh [17] found that the ITZ in concrete made with recycled fine aggregates (RFA) also affects durability, particularly in relation to chloride penetration. Furthermore, Barabanshchikov et al. [18] focusing on high-calcium fly ash aggregates, confirmed that while high strength can be achieved, their susceptibility to factors like chloride ingress and freeze–thaw damage remains a key barrier to wider structural application. Moreover, Xu et al. [19] focused on nanomaterials to enhance the ITZ but noted that little research explores the combined effects of gypsum activation and carbonation curing on ITZ properties, particularly in sulfur-rich systems like CFBFA. This study aims to investigate the synergistic effects of gypsum activation and carbonation curing in improving ITZ characteristics and concrete performance, filling a gap in existing research.

Carbonation curing has been widely studied for improving the properties of artificial aggregates. For instance, carbonation during post-curing has significantly enhanced the strength of steel-making slag aggregates by 220% [20]. Building on this, Xu et al. [21] showed that flue gas carbonation can improve the strength and microstructure of CFBFA aggregates. Mehmood et al. [22] and Liu et al. [23] confirmed the efficacy of CO_2_ enhancement in recycled aggregates; they observed that high-pressure carbonation increases CaCO_3_ formation, which densifies the adhered mortar, effectively filling the ITZ and reducing crack width. Despite these advancements, the effects of carbonation curing on the interfacial transition zone (ITZ) in CFBFA-based aggregates remain underexplored. This study aims to address this gap by investigating how gypsum activation and carbonation curing impact ITZ properties, offering insights into improving concrete performance using industrial by-product-based aggregates.

Non-contact measurement methods, particularly those based on image processing, offer significant advantages in precision and efficiency. For example, Zhang et al. [24] developed a contact-free method using gray-scale analysis to measure the thickness of oxide layers. This technique, combined with XPS analysis, enabled a rapid and accurate determination of layer thickness while being both cost-effective and highly efficient. Similarly, Hu et al. [25] proposed a method that uses image processing and a binarization algorithm to measure the thickness of ice on power lines and insulators. Despite the success of these methods in measuring other material surfaces, their application to the interfacial transition zone (ITZ) of concrete remains limited. This is especially true for concrete made with circulating fluidized bed fly ash (CFBFA) artificial aggregates. Due to the secondary hydration properties of CFBFA, the ITZ is a dynamic and complex region, making traditional measurement methods largely unsuitable.

Despite notable progress in individual strategies such as gypsum activation and carbonation curing, their synergistic effects on the interfacial transition zone (ITZ) of CFBFA-based artificial aggregates remain poorly understood. For instance, Wang et al. [26] recently demonstrated that carbonate can synergistically stabilize AFt crystal growth, which enhances the mechanical performance of cementitious matrices. Gypsum activation facilitates early ettringite (AFt) formation, enhancing particle bonding and microstructure densification, while carbonation curing promotes calcium carbonate (CaCO_3_) precipitation, filling microcracks and further refining the ITZ. However, few studies have addressed how these two mechanisms interact in sulfur-rich, pozzolanic systems like CFBFA, particularly in the presence of secondary hydration. Moreover, traditional ITZ assessment techniques often lack the resolution and objectivity needed for accurate characterization in such complex matrices.

To bridge this gap, this study introduces a novel dual-enhancement strategy combining gypsum activation with pressurized flue gas heat curing (FHC), a technique that leverages both CO_2_ and thermal energy from industrial emissions. This environmentally friendly curing approach enhances aggregate quality while contributing to carbon capture. In parallel, a non-contact grayscale image analysis method is proposed to quantitatively evaluate ITZ thickness and morphology, offering a precise, reproducible, and cost-effective alternative to conventional methods.

This research systematically investigates the influence of combined FHC and gypsum activation on the ITZ and macro-scale performance of concrete containing over 80% CFBFA-based artificial aggregates. The main objectives are to reveal the synergistic mechanisms by which gypsum-induced AFt and FHC-induced CaCO_3_ cooperatively refine the ITZ microstructure; establish quantitative correlations between ITZ characteristics and concrete properties; and identify the shift in failure mode from interface-dominated to composite-controlled behavior, indicating successful ITZ optimization. In summary, while previous studies have independently explored gypsum activation or carbonation curing, the combined effects of these two strategies—particularly in sulfur-rich, low-reactivity systems like CFBFA—remain underexplored. Furthermore, quantitative links between ITZ microstructure and macro-scale mechanical behavior are rarely addressed using high-resolution and non-invasive techniques. To bridge these gaps, this study introduces a dual-enhancement approach, integrating gypsum-induced ettringite formation with pressurized flue gas carbonation, aiming to synergistically improve ITZ structure and mechanical performance. In parallel, the application of a grayscale-based image analysis method offers a novel, cost-effective, and reproducible tool for precise ITZ evaluation. By achieving these aims, the study contributes a robust framework for optimizing low-carbon cementitious materials based on industrial by-products, offering both scientific insights and practical pathways toward greener and more durable construction.

## 2. Materials and Methods

### 2.1. Materials and Mix Design

Portland cement (P.O. 42.5), circulating fluidized bed fly ash (CFBFA), gypsum (CaSO_4_·2H_2_O; CAS 10101-41-4, 99%), standard sand, polycarboxylate ether (PCE), and hydrated lime (Ca(OH)_2_; CAS 1305-62-0, 95%) were used as raw materials. CFBFA was supplied by Shanxi Jinneng Datuhe Thermal Power Co., Ltd. (Lüliang, China), while the Portland cement (P.O. 42.5) was from Jiuqi Building Materials Co., Ltd. (Zhucheng, China). Gypsum was purchased from Shanghai Aladdin Biochemical Technology Co., Ltd. (Shanghai, China), and hydrated lime was from Shanghai Macklin Biochemical Technology Co., Ltd. (Shanghai, China) A commercially available liquid polycarboxylate ether (PCE) superplasticizer was obtained from a local supplier (Taiyuan, China). The standard sand used was procured from Xiamen ISO Standard Sand Co., Ltd., Xiamen, China, with an average particle diameter (D50) of 0.17 mm. The physical properties of the standard sand used in this study are characterized through a series of laboratory tests conducted in accordance with the Chinese national standard GB/T 50123-2019 [27]. Based on the average of three replicate measurements (N = 3), the sand exhibits a specific gravity (G_s_) of 2.63 (SD = 0.013), a maximum void ratio (e_max_) of 0.95 (SD = 0.015), and a minimum void ratio (e_min_) of 0.61 (SD = 0.019). Sieve analysis reveals that the majority of particles (64.73%) fall within the 0.1–1.0 mm size range, while 33.55% are between 1.0 and 2.0 mm. Particles between 0.075 mm and 0.1 mm account for 1.64% of the total mass, and the fines content (<0.075 mm) is negligible at 0.08%. Replicate tests confirm this particle distribution with high consistency, as all reported standard deviations are below 0.5%. These results confirm that the material is a clean, well-graded sand, suitable for use in standardized concrete applications.

**Figure 1:**
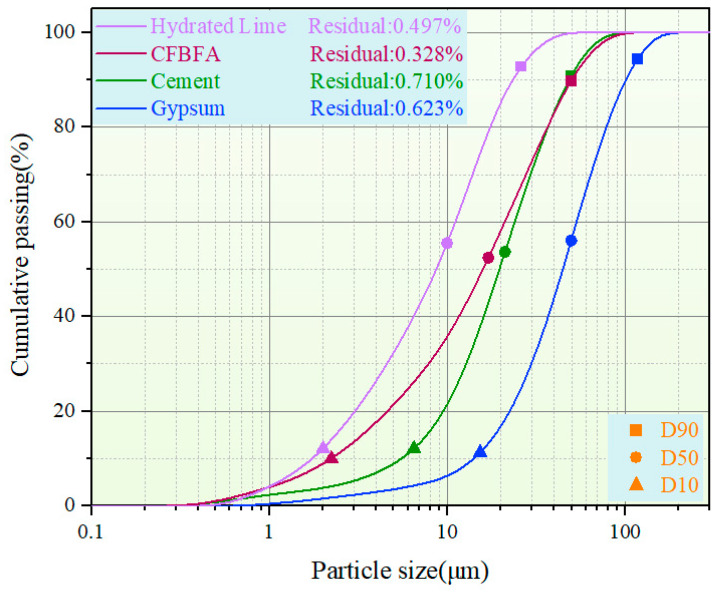
Particle size distribution of the raw materials measured by laser granulometry.

The chemical compositions of CFBFA, Portland cement, and hydrated lime were determined using X-ray Fluorescence (XRF) (Bruker, Karlsruhe, Germany). The results are presented in Table 1. The particle size distributions of these materials were measured with a laser particle size analyzer (BT-9300HT, Bettersize, Costa Mesa, CA, USA), with results shown in Figure 1. According to these measurements, the specific surface areas of CFBFA, cement, and hydrated lime were 333.6, 213.0, and 974.5 m^2^/kg, and their median diameters (D50) were 16.98, 21.89, and 9.663 µm, respectively.

**Figure 2:**
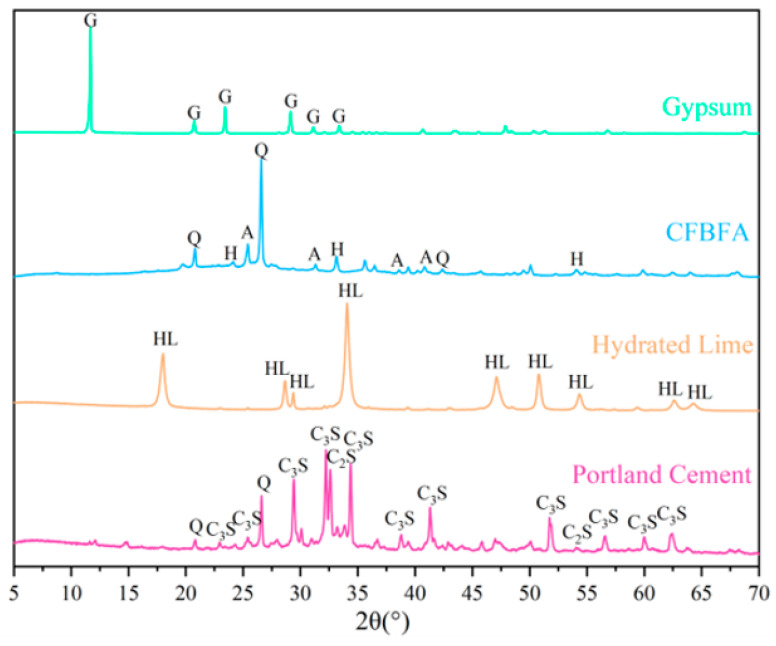
XRD pattern for raw materials. (Q: Quartz (46-1045) [28]; H: Hematite (33-664) [29]; and G: Gypsum (JCPDS 33-0311 [30]); A: Anhydrite (37-1496); HL: Hydrated Lime (4-733 [31]); C_3_S: Tricalcium Silicate (JCPDS 42-0551) [32]; C_2_S: Dicalcium Silicate (33-0302) [32]).

The crystalline phases of the raw materials were identified using a Rigaku Ultima IV diffractometer (40 kV, 40 mA) with a 2θ scanning range of 5–70°, a step size of 0.02°, and a scanning speed of 3°/min. The XRD patterns are shown in Figure 2. All XRD measurements were performed in triplicate, and the figure shows the representative pattern due to excellent repeatability. Crystalline phases were identified by comparing the collected diffraction data with the JCPDS standards database, and the specific JCPDS card numbers used for phase identification are detailed in the figure caption. The primary crystalline phases in CFBFA were Quartz (SiO_2_), Anhydrite (CaSO_4_) and Hematite (Fe_2_O_3_). For cement, the main phases were Quartz (SiO_2_), Hatrurite (C_3_S), and Larnite (C_2_S).

All concrete mix proportions and process parameters were kept consistent, with the exception of the coarse aggregate type. The mix proportions of the reference concrete are detailed in Table 2. The four concrete mixes were designed solely by replacing the artificial coarse aggregates with the different types listed in Table 3. The mass of the coarse aggregate was adjusted according to its bulk density to maintain a constant solid volume. The physical properties of the aggregates are summarized in Table 4.

### 2.2. Preparation of Artificial Aggregates and Concrete Specimen

**Figure 3:**
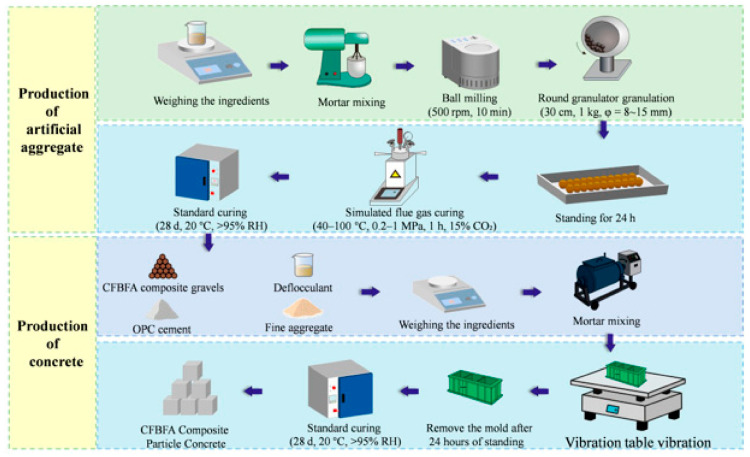
Preparation process of CFBFA composite gravel concrete.

The preparation of artificial aggregates and concrete specimens is shown in Figure 3.

1. Preparation of CFBFA Artificial Aggregates (Composite Gravels)

First, the dry raw materials were weighed and mixed into a mortar. This mixture was subjected to ball milling for 10 min at 500 rpm. The milled material was then fed into a round granulator to form composite gravels—our term for the CFBFA-based artificial aggregates—with a diameter of 8–15 mm. These aggregates were left to stand for 24 h.

After standing, the composite gravels were divided into two groups for different curing methods: Standard Curing: One group was cured for 28 days in a climate chamber at a temperature of 20 °C and a relative humidity (RH) of over 95%. Simulated Flue Gas Curing: The other group was placed in a pressure vessel for 1 h, where they underwent curing with simulated flue gas at a fixed temperature of 80 °C, a pressure of 1 MPa, and a CO_2_ concentration of 15%. This group was then transferred to standard curing conditions for the remainder of the 28-day period.

2. Concrete Mixing and Curing

The prepared CFBFA composite gravels were used to make concrete. The aggregates, OPC cement, fine aggregate, and water reducer were weighed according to the mix proportions. These ingredients were then mixed into a fresh concrete mortar. The mortar was poured into molds and placed on a vibration table to ensure proper compaction. After standing for 24 h, the molds were removed, and the concrete specimens were placed in the same standard curing environment as the aggregates for 28 days.

### 2.3. Testing and Characterization

The unconfined compressive strength (UCS) of concrete samples was determined following the general principles of ASTM C39 [33]. The water absorption and bulk density of concrete samples were determined following the general principles of ASTM C642 [34]. The determination of Young’s Modulus is in accordance with ASTM E1876-22 [35]. The microstructure was analyzed via scanning electron microscopy SEM using a JEOL JSM-IT200 (JEOL Ltd., Tokyo, Japan). SEM analyses were conducted to evaluate the microstructural evolution related to the curing strategies. For SEM, block-shaped samples were selected, and a conductive metal coating was applied to enhance imaging quality. To investigate the interfacial microstructure evolution, three distinct sampling regions were selected from the fractured concrete specimens: (i) Aggregate core: the central portion of a coarse aggregate exposed after UCS failure; (ii) ITZ region: including aggregate–paste interface; (iii) Matrix zone: paste segments at least 5 mm away from any visible aggregate surface. All samples were vacuum-dried at 40 °C and coated with a 10 nm layer of gold prior to SEM analysis.

### 2.4. Image Analysis for ITZ and Statistics

**Figure 4:**
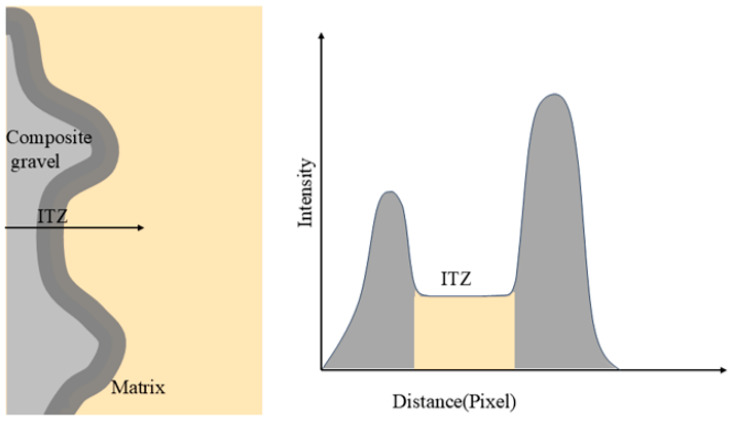
Quantitative analysis of ITZ thickness in composite gravel using double-peak gradient technique.

The thickness of the interfacial transition zone (ITZ) was quantitatively measured from SEM images using Image-Pro Plus 6.0 (Media Cybernetics, Rockville, MD, USA) combined with a custom post-processing procedure. Grayscale line profiles were first extracted perpendicular to the aggregate–matrix interface using Image-Pro Plus 6.0 with a scale-calibrated pixel–length conversion. These profiles were then processed using a MATLAB R2021b (MathWorks, Natick, MA, USA) script implementing a dual-peak gradient algorithm for automatic ITZ width quantification. Each profile was smoothed to minimize image noise, and the absolute first derivative of grayscale intensity was computed. The two most prominent gradient peaks, typically located within the outer thirds of the profile, were automatically identified and interpreted as the ITZ boundaries. The pixel distance between these peaks was converted into microns according to the calibrated scale bar (Figure 4). For each mixture, 20 linear profiles were analyzed at different cross-sectional locations to ensure statistical representativeness. The reported ITZ thickness values are expressed as the arithmetic mean ± standard deviation, providing a quantitative basis for comparing ITZ development under different curing regimes and gypsum contents.

## 3. Result and Discussion

### 3.1. Physical Properties

**Figure 5:**
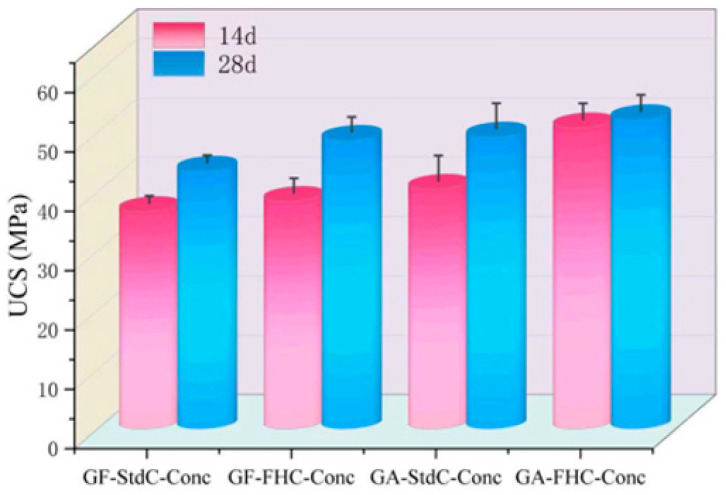
Unconfined compressive strength (UCS) of concretes at 14 and 28 d.

**Figure 6:**
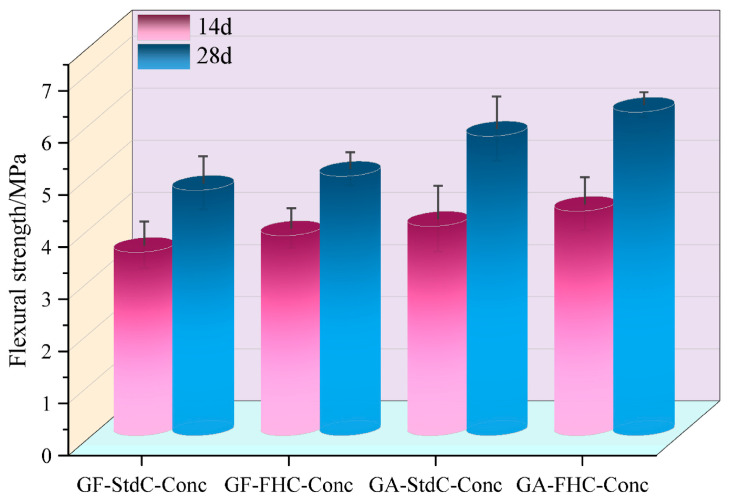
Flexural strength of concretes at 14 and 28 d.

**Figure 7:**
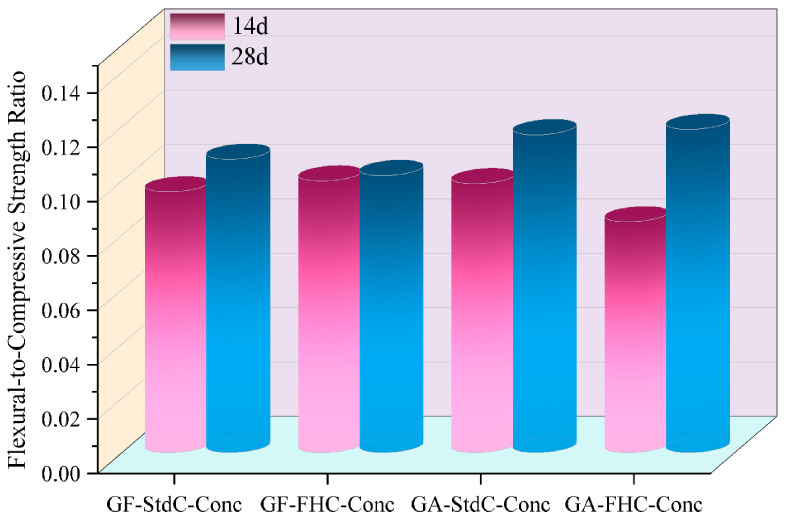
Flexural-to-compressive strength ratio of concretes at 14 and 28 d.

Figure 5 illustrates the unconfined compressive strength (UCS) of concretes at 14 and 28 days. A consistent hierarchy is observed at both ages: GF-StdC-Conc < GF-FHC-Conc ≈ GA-StdC-Conc < GA-FHC-Conc. Thus, carbonation and gypsum addition each raise strength relative to the gypsum-free, non-carbonated baseline, and their combination gives the highest UCS. From 14 to 28 d all mixes gain strength. Figure 6 shows flexural strength increased for all groups from 14 d to 28 d. The benchmark GF-StdC-Conc exhibited the lowest strength at 28 d. The GF-FHC-Conc group showed a slight increase, while the GA-StdC-Conc group showed a more significant increase by 28 d. The GA-FHC-Conc group displayed the best performance, showing high strength at 14 d and achieving the greatest overall improvement by 28 d. Figure 7 shows the flexural-to-compressive strength (F/C) ratio, which generally trended upward from 14 d to 28 d. At 28 d, the gypsum-added (GA) groups exhibited superior F/C ratios to the non-gypsum groups, suggesting improved toughness. The GA-FHC-Conc group had a low initial ratio at 14 d, attributed to its high early compressive strength. However, its ratio rose significantly by 28 d—the highest of all groups—reflecting a balanced improvement and optimal toughness.

**Figure 8:**
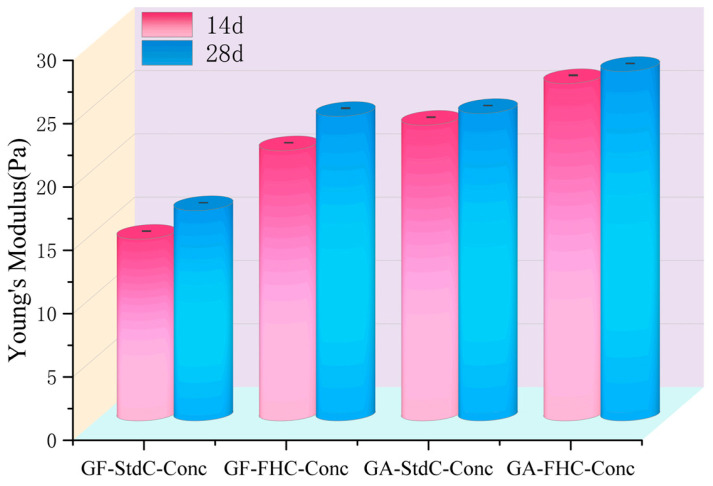
Young’s Modulus of concretes at 14 and 28 d.

Figure 8 compares the Young’s modulus of concretes made with different aggregate types and curing regimes at 14 and 28 days. GA-FHC-Conc exhibited the highest modulus at both curing ages, indicating superior stiffness. Generally, concretes incorporating carbonated aggregates outperformed those using non-carbonated aggregate. The results demonstrate that both aggregate nature and curing method critically influence elastic behavior, with FHC promoting early-age stiffness and continuous modulus development over time.

**Figure 9:**
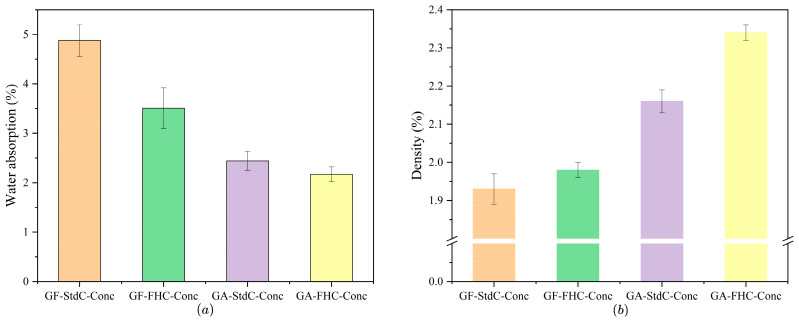
Physical properties of concretes: left (**a**) water absorption; right (**b**) bulk density.

Figure 9 presents the physical properties of the concretes, specifically (a) water absorption and (b) bulk density. Water absorption exhibits a monotonic decrease from GF-StdC-Conc to GF-FHC-Conc to GA-StdC-Conc to GA-FHC-Conc. Thus, both carbonation and gypsum addition reduce the accessible pore volume, with gypsum providing the larger primary drop and carbonation delivering a secondary reduction. This ranking mirrors the mechanical hierarchy (UCS, E), suggesting that lower uptake is directly associated with improved load-bearing capacity. Bulk density follows the reverse trend of absorption and increases in the same sequence (GF-StdC < GF-FHC < GA-StdC < GA-FHC), evidencing progressive densification of the hardened microstructure. The density gain imparted by gypsum is substantial, and carbonation further elevates density, especially when gypsum is present.

### 3.2. ITZ Thickness Analysis

The ITZ thickness was estimated from SEM grayscale profiles using a dual-gradient peak method. Each profile was smoothed, and the two most prominent gradients—interpreted as ITZ boundaries—were detected. The pixel distance was converted to microns using a calibrated scale bar. Batch processing was performed via custom MATLAB R2021b scripts.

**Figure 10:**
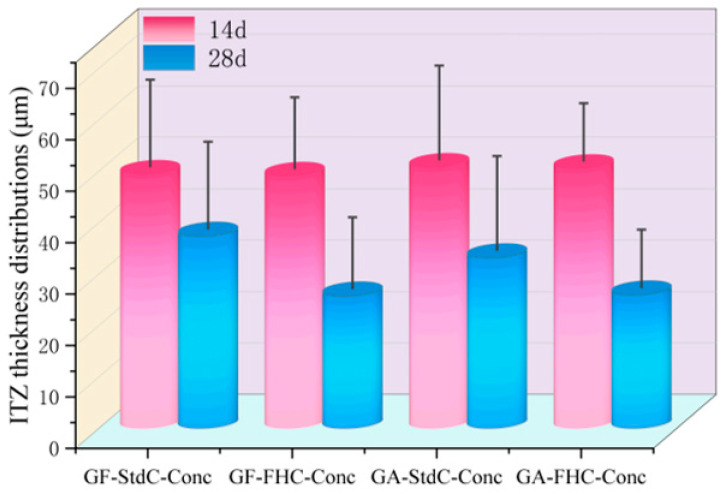
Interfacial transition zone (ITZ) thickness distributions at 14 and 28 d.

As can be seen from Figure 10, cross all mixes, the ITZ thickness at 14 d is essentially similar. Thus, neither gypsum addition nor carbonation produces a measurable refinement at early age within the experimental scatter.

By 28 d, all mixes display a clear reduction, with the carbonated concretes showing the largest refinement. Curing refines the ITZ, but carbonation dominates the refinement at 28 d—carbonated mixes stabilize near 26 µm, about 9–11 µm thinner than their non-carbonated counterparts. The scatter narrows at 28 d, indicating a more homogeneous interphase as hydration and carbonation proceeds.

**Figure 11:**
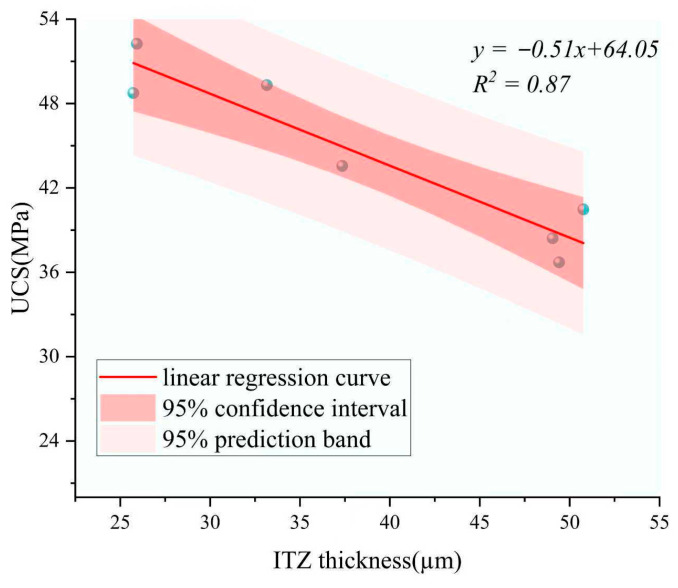
Regression between ITZ thickness and UCS.

As shown in Figure 11, a strong negative linear correlation was observed between ITZ thickness and UCS, with a coefficient of determination (R^2^) of 0.87. Both the 95% confidence interval and prediction band are included to enhance the statistical robustness of the regression analysis. This indicates that a thicker ITZ is generally associated with reduced compressive strength, likely due to weaker bonding and increased porosity at the interface. The narrow confidence interval and prediction band further support the robustness of this trend. To better capture the representative trend among typical samples, one data point with poor linear fitting performance was excluded from the regression analysis. To ensure the robustness of the ITZ–UCS regression analysis, one data point corresponding to the 14-day GA-FHC-Conc sample was excluded from the fitting. This sample exhibited an unusually high compressive strength despite its relatively wide ITZ, deviating significantly from the overall trend. This anomaly may be attributed to the early-age microstructural densification induced by the synergistic action of flue gas carbonation and gypsum activation, which compensated for the otherwise detrimental effect of a thicker ITZ. As the objective of the regression is to reveal the general correlation between ITZ thickness and mechanical behavior, removing this statistical outlier allowed for a clearer trendline and improved model accuracy. This point exhibited noticeable deviation from the general pattern. Using the remaining seven data points, a clear inverse relationship between ITZ thickness and crushing strength was observed, indicating that thinner ITZ regions tend to correspond to higher mechanical performance.

### 3.3. SEM Microstructure

**Figure 12:**
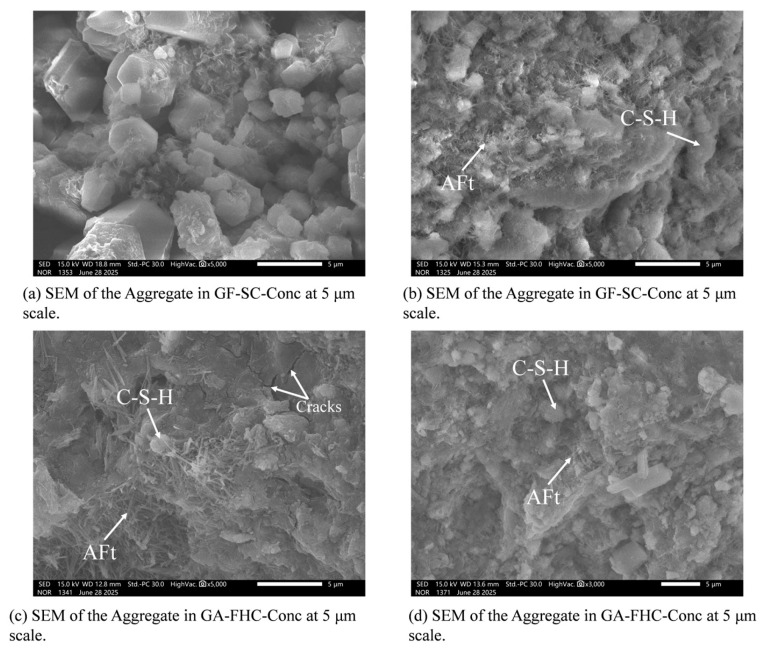
Aggregate region microstructure at 14 d.

Figure 12 presents the aggregate region microstructure at 14 d. Figure 12a shows abundant angular CFBFA particles with sharp edges; inter-particle gaps are evident and only sparse adherent hydrates are present. Narrow constrictions between adjacent pores form apparent pore throats in the 2D SEM view; here, “pore throat” denotes the narrowest inter-pore constriction observed in 2D and does not quantify three-dimensional connectivity [36]. The overall fabric is therefore loose with weak gel connectivity. Figure 12b shows that carbonation yields smoother and more continuous pore walls with narrowed inter-pore constrictions, implying finer and more uniformly distributed hydration products and a clear densification of the aggregate interior [37,38]. Figure 12c shows that in the gypsum-added, non-carbonated mix, needle-like AFt crystallites preferentially grow in the pores; the cracks marked in the panel were induced by UCS loading. Figure 12d shows that, relative to Figure 12c, hydration products are shorter, finer, and more uniformly distributed, frequently embedded within C–(A)–S–H gel. Overall, carbonation produces a denser and more homogeneous morphology within the aggregate.

**Figure 13:**
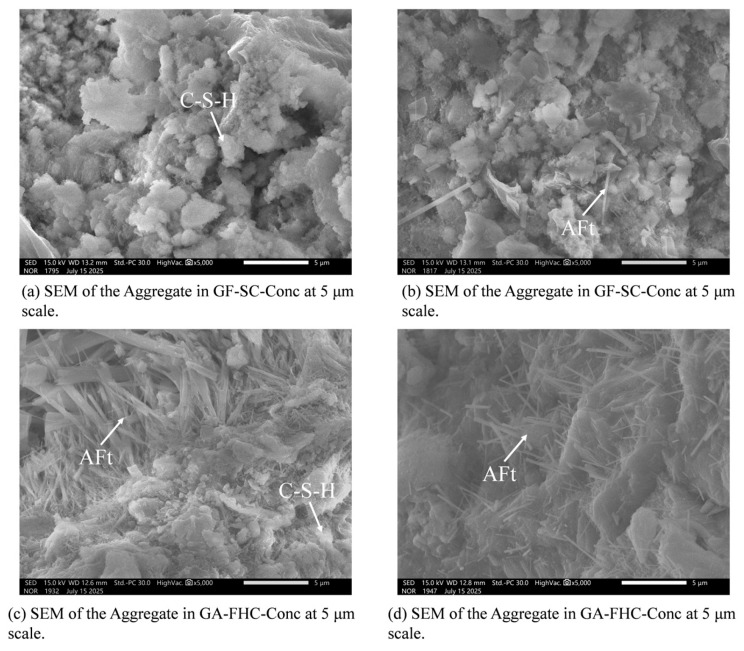
Aggregate region microstructure at 28 d.

Figure 13 presents the aggregate-region microstructure at 28 d. Figure 13a illustrates a rough microstructure, characterized by relatively open pores and a loosely packed, non-integrated texture. Compared to the 14-day sample, a greater quantity of hydration products is observed, indicating continued secondary hydration within the aggregate between 14 and 28 days. As shown in Figure 13b, carbonation leads to product refinement and morphological densification [39,40]. The 28-day microstructure (Figure 13c) consists of large clusters of strongly oriented, needle-like hydration products (AFt). The sample (Figure 13d) reveals slightly thicker but more uniformly distributed AFt crystals embedded within a more continuous C–S–H gel. This progression yields an overall denser and more homogeneous aggregate morphology.

**Figure 14:**
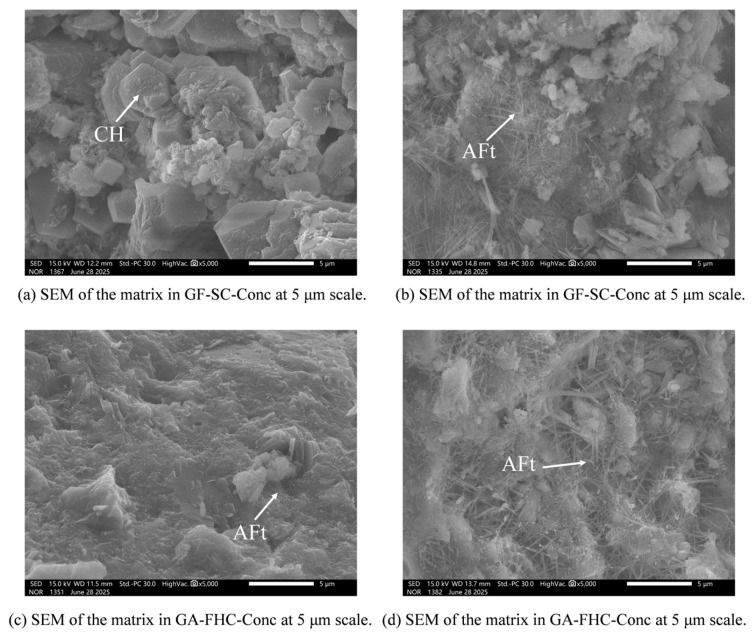
Matrix region microstructure at 14 d.

Figure 14 presents the matrix-region microstructure at 14 d. Figure 14a shows a relatively rough matrix with flaky or plate-like hydrates and scattered unreacted residues [41,42], indicating limited gel connectivity and an immature hydration structure. Figure 14b shows abundant needle-like AFt crystals within the matrix. This AFt is interpreted as a result of sulfate released from the aggregate. Figure 14c exhibits a denser matrix with better gel continuity. Figure 14d shows thicker yet more uniformly distributed AFt needles, largely embedded within a continuous C–(A)–S–H gel phase. The more even distribution of AFt is consistent with a carbonate shell on the aggregate that buffers sulfate release, ultimately yielding a denser and more homogeneous matrix.

**Figure 15:**
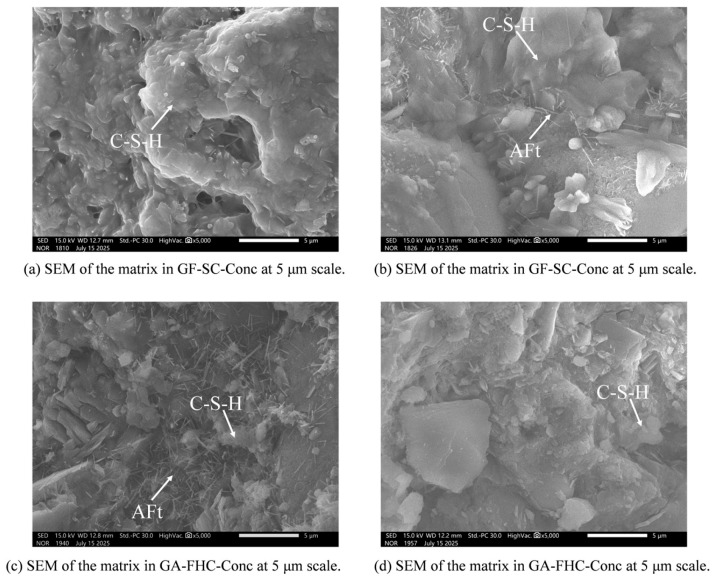
Matrix region microstructure at 28 d.

Figure 15 presents the matrix-region microstructure at 28 d. Figure 15a shows a rough matrix with locally open pores, indicating limited densification compared to the 14 d state. Figure 15b displays a few slender AFt needles interwoven with C–(A)–S–H gel. Figure 15c exhibits abundant needle- and bundle-like hydrates intertwined with C–(A)–S–H gel. Figure 15d reveals the densest and most homogeneous matrix among all samples: the AFt crystals are shorter and more evenly distributed, largely embedded within a continuous gel phase.

Overall, gypsum promotes AFt formation within the matrix, while carbonation of the aggregate refines and homogenizes the hydrate assemblage via ITZ-mediated effects. This combined condition results in the most compact and uniform microstructure, consistent with the observed reductions in water absorption and improvements in bulk density, UCS, and Young’s modulus.

**Figure 16:**
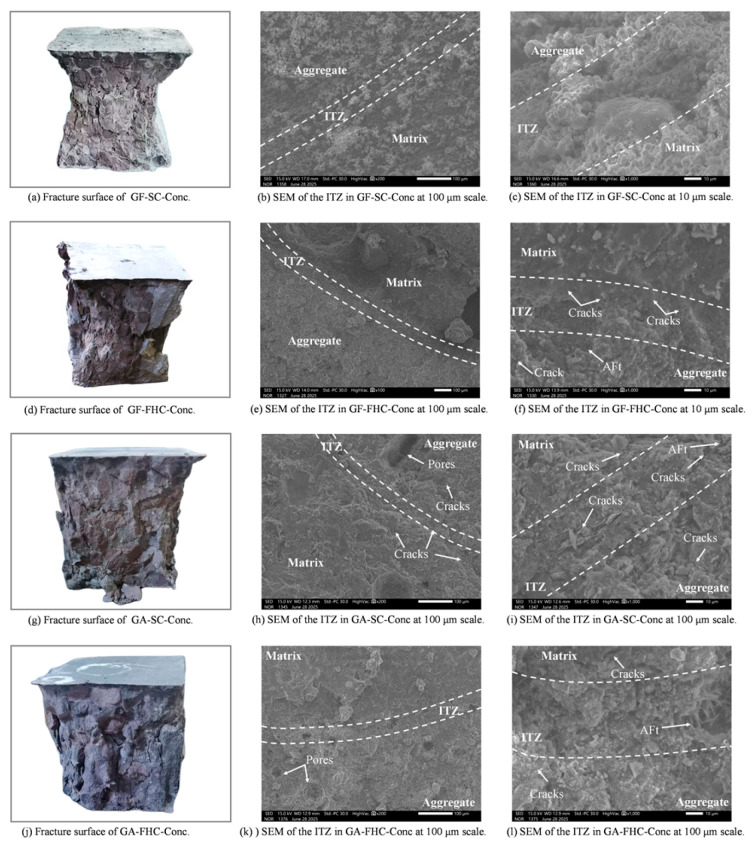
ITZ microstructure at 14 d.

Figure 16a shows the fracture morphology of GF-SC-Conc after compressive failure at 14 days. The overall fracture surface exhibits a distinctly rough “tearing–pull-out” composite failure mode. Multiple aggregate particles appear to have been pulled out, forming concave, pit-like spalling zones, indicating that the aggregate–matrix interface (ITZ) served as the primary fracture path [43]. The clearly defined contours of the aggregates suggest a weak ITZ strength and insufficient interfacial bonding in this system. Crack propagation mainly follows the boundaries of the aggregates, reflecting a prominently interface-controlled failure mode, with no evidence of effective shear bridging or matrix interlock [44]. In some areas, deep traces of detached aggregates are visible, suggesting that interfacial microcracks may have already initiated and penetrated prior to loading, indicating limited overall crack resistance. This fracture morphology reveals that, during early hydration, the GF-SC-Conc system lacked sulfate-assisted reactions and structural densification, resulting in sparse and weak interfacial products. Consequently, failure primarily occurred along the aggregate interface, manifesting as a “aggregate debonding and ITZ failure” mode at the macroscopic level. Corresponding to Figure 16b,c, the SEM images show the interfacial transition zone (ITZ) at 14 days. The ITZ region exhibits low contrast and a loose structure. Unreacted residues of CFBFA remain visible at the aggregate edges, with a rough particle surface, indicating a lack of sulfate-induced activation from gypsum. As a result, secondary hydration at the interface is significantly limited. In Figure 16b, the ITZ appears relatively wide and lacks dense filling products, with only sparse plate-like hydrates observed. Figure 16c, at a higher magnification, reveals that the matrix side mainly consists of coarse C–S–H, while no evident interlocking structure is found at the aggregate boundary. Overall, the ITZ is still in an early stage of development, with blurred boundaries and no apparent interpenetration structure, making it a preferred path for stress concentration and crack propagation. Combining macro- and micro-level observations, it can be concluded that the fracture behavior of the GF-SC-Conc system at 14 days is closely related to the underdeveloped interfacial structure. Due to the low reactivity on the aggregate side and the absence of sulfate-induced encapsulation, strong interfacial bonding fails to form, leading to a brittle failure mode dominated by aggregate debonding and ITZ cracking.

GF-FHC-Conc corresponds to the carbonated, gypsum-free system after 14 days of curing. As shown in the macroscopic image (Figure 16), its fracture morphology differs significantly from that of the uncarbonated GF-SC-Conc specimen. The fracture surface appears smoother and denser, with markedly fewer spalling pits. The exposed aggregate surfaces are relatively clean, and in some regions, signs of aggregate rupture can even be observed—suggesting that the interfacial strength has increased to a level comparable to the aggregate itself, causing cracks to penetrate through the matrix or aggregates. The aggregate boundaries are no longer sharply defined but appear rounded and passivated, likely due to the formation of a carbonate reaction shell on the aggregate surface. This shell may contribute to improved microstructural morphology and reduced local stress concentration. The fracture path no longer follows the aggregate boundary exclusively, but instead cuts through the matrix or bends deeply along the interface [45], indicating enhanced overall bonding performance. The failure mode thus transitions from interface-dominated to a more complex, composite fracture pattern [44]. The macroscopic observations align well with the SEM results. As shown in Figure 16e, the ITZ at 14 d exhibits a well-defined boundary, with a continuous and compact structure formed between the aggregate and the matrix. At higher magnification (Figure 16f), the interface reveals short, bundled needle-like products—identified as refined AFt and C–(A)–S–H gels—distributed in a bridging and interwoven pattern along the ITZ. In some regions, tangential microcracks are visible along the interface, but they remain unpenetrated, indicating that crack initiation is effectively suppressed. These microstructural characteristics suggest that the carbonate shell formed during carbonation serves a dual function at the aggregate surface: it acts as a nucleation site for hydration products and simultaneously as a diffusion barrier. This dual role facilitates the uniform precipitation of refined C–(A)–S–H and AFt phases, while effectively reducing porosity and interrupting microcrack pathways at the interface. As a result, the interfacial transition zone (ITZ) becomes denser due to enhanced interfacial bonding. Moreover, the shell’s buffering effect on SO_4_^2−^ ionic flux facilitates the formation of finer, more uniform hydration products at the interface, avoiding localized supersaturation and the formation of large crystalline clusters. This refined filling reduces stress concentrations and helps to evenly distribute fracture energy, further enhancing the mechanical integrity of the composite.

These microstructural features are consistent with the observed reduction in ITZ thickness, increase in elastic modulus (E), improvement in UCS, and decrease in water absorption rate, indicating that carbonation treatment can significantly enhance interfacial performance even in gypsum-free environments. This shell-based mechanism offers a feasible strategy for interfacial regulation in CFBFA-based aggregates under sulfate-deficient conditions, thereby supporting their broader engineering application.

Figure 16g illustrates the fracture surface of the GA-SC-Conc specimen, exhibiting a distinct “interfacial debonding” failure mode. Key observations include: the overall fracture surface is rough and fragmented, with widespread impressions of multiple intact aggregate outlines remaining visible; multiple branched cracks appear in localized regions, indicating inadequate ITZ strength, causing cracks to “detour” along the interface rather than penetrate through it. Overall, the failure reflects a “interface-dominated with partial matrix cracking” composite fracture mode.

Combined with SEM images Figure 16h,i, it can be deduced that in the non-carbonated system, although the introduction of gypsum promotes AFt formation, the resulting crystals tend to be coarse, loosely packed, and accompanied by connected microcracks. This creates a weak ITZ structure characterized by “local porosity”, making the interface the primary zone of failure.

In Figure 16g, microcracks are observed at the interface and aggregate side in the UCS-fractured GA-SC-Conc specimen. In contrast, the ITZ of GA-FHC-Conc appears denser and more continuous, with almost no visible interfacial cracks after UCS failure. This suggests a more effective distribution of micro-scale bridging across the interface and reduced local stress concentrations. The observed difference aligns with the hypothesized effects of carbonation curing, including ion flux homogenization and delayed release. In stark contrast to Figure 16g, this fractured surface exhibits a more compact, coherent, and structurally defined morphology. The overall surface is flatter and more continuous, with significantly fewer signs of debonding, indicating a notable enhancement in the bond strength between aggregate and matrix. Fracture traces around aggregate edges appear “passivated,” lacking sharp spalling or detachment, suggesting that the carbonation-induced shell provided a restraining effect at the aggregate surface. Some aggregates appear to fracture internally, or the crack paths extend through the matrix region—implying that the interfacial strength is no longer the dominant weakness. This fracture morphology confirms that the combined action of carbonation and gypsum results in carbonated shell-assisted pore closure, and reduced crack connectivity. Together, these mechanisms shift the failure path away from the interface toward the composite matrix/aggregate region, forming a typical “structurally integrated, interface-stabilized” failure mode [46].

**Figure 17:**
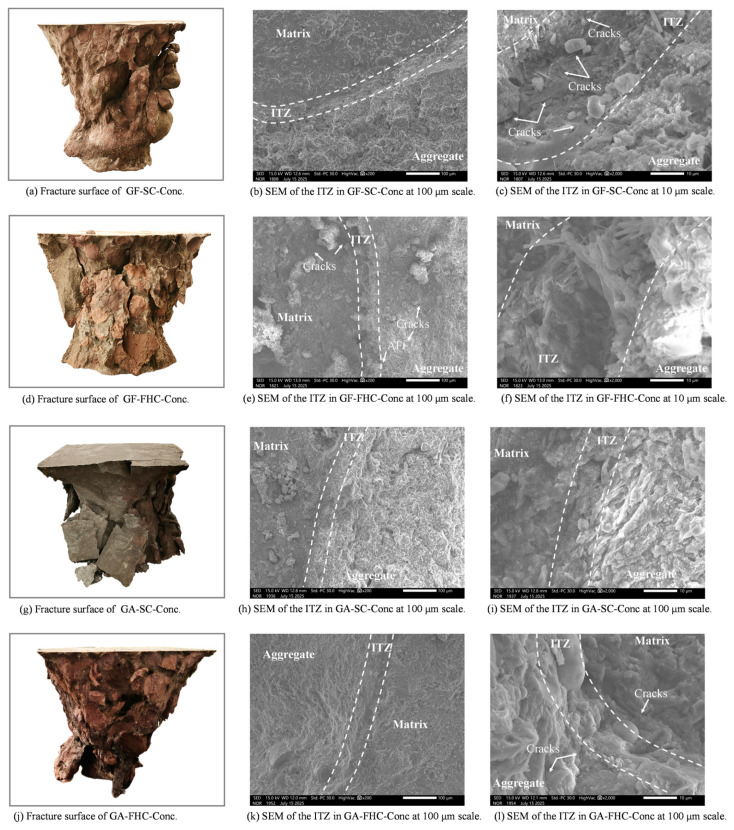
ITZ microstructure at 28 d.

As shown in Figure 17a, the GF-SC-Conc sample exhibits a typical “aggregate pull-out and surface fragmentation” composite failure mode under compressive loading. The fracture surface is generally rough, with clearly exposed and intact aggregate outlines and minimal matrix adhesion, indicating weak interfacial bonding. Cracks preferentially propagate along the aggregate–matrix interface (ITZ) rather than through the aggregates or matrix itself. In localized areas, severe fragmentation and dense cracking are observed, with visible signs of layered spalling along the aggregate distribution, suggesting rapid microcrack accumulation and penetration under loading. The corresponding Figure 17b,c reveal a porous and loosely structured ITZ. Multiple fine cracks are seen propagating along the interfacial zone, confirming the ITZ as the preferred crack propagation path. Compared with the 14-day samples, the ITZ in the image shows a certain degree of densification on both the matrix and aggregate sides: CH is significantly reduced, C–S–H gel becomes more abundant. However, compared with other mix designs, this sample still exhibits a relatively thick ITZ with non-uniform product distribution. This indicates that the interfacial zone remains a primary site for stress concentration and crack propagation. The initially thick ITZ formed at early ages, with unsealed micropores and capillary channels; the slow secondary hydration between SiO_2_/Al_2_O_3_ in the aggregate and CH in the matrix continues. However, even at 28 days, the early-formed ITZ and its interconnected pores have not been fully passivated. This is consistent with the relatively low UCS and Young’s modulus observed for this mix, revealing a structural defect evolution path of “crack propagation along the ITZ.”

Figure 17d shows the compressive fracture surface of the GF-FHC-Conc specimen after 28 days of carbonation curing. Compared with the fragmented morphology of GF-SC-Conc in Figure 17a, this specimen exhibits a denser, more regular fracture surface with clearer contours and relatively flat planes, indicating improved structural integrity and enhanced interfacial bonding. Most aggregates fractured through shear failure rather than complete pull-out, suggesting a significant improvement in ITZ bonding strength. Small amounts of matrix still adhere to the aggregate surfaces, and the contours appear less sharp, reflecting that the interface is no longer the preferred crack propagation path [47]. The enhanced interface bonding and the tendency of cracks to deviate from the interface confirm the densification and crack-deflection mechanisms induced by carbonation. In the corresponding Figure 17e,f, the ITZ exhibits significant densification and clearly defined boundaries. The AFt crystals are more finely and orderly distributed. Interfacial cracks are mostly oriented tangentially with short propagation distances, indicating a certain degree of crack passivation. This aligns well with the “non-interface-dominated” failure pattern observed in the macroscopic fracture surface. As shown in Figure 17e, the GF–FHC-Conc specimen at 28 days developed a carpet-like deposition of fine needle-like structures along the ITZ, effectively “structurally stitching” the aggregate to the matrix. These changes collectively drove the ITZ to evolve from a thick and loose early-stage structure to a denser and more stable one at later ages, reducing crack concentration and promoting curved or deflected propagation paths. During carbonation, the formation of a CaCO_3_ shell layer served both as a heterogeneous nucleation site and as a flux equalizer for SO_4_^2−^ migration. Together, these effects encouraged the interface products to spread uniformly along the ITZ, forming distributed micro-bridging structures composed primarily of refined AFt and C–(A)–S–H gels. This mechanism achieved effective filling without inducing excessive expansion. In summary, carbonation significantly optimized the ITZ structure and crack behavior in gypsum-free systems, shifting the failure mode from “interface dominated” to “non-interface-dominated,” thereby laying a microstructural foundation for enhanced structural stability.

As shown in Figure 17g the fracture morphology typically exhibits an interface-dominated failure mode. The overall fracture surface is highly fragmented, with clear separation between aggregates and matrix. Corresponding to Figure 17h,i, the ITZ displays pronounced grayscale contrast, with large, loosely packed AFt crystals and densely scattered, disordered microcracks, supporting the macroscopic observation of a “multi-point destabilization and interfacial tearing” failure mode [48,49]. Compared to the 14-day specimens, the ITZ band in GA-SC-Conc-28d becomes overall denser and more compact. On the matrix side, a greater coexistence of AFt and C–(A)–S–H gel can be seen, with a reduced grayscale difference between the matrix and the interface. However, the aggregate–matrix boundary remains clearly visible, indicating that the continuity and bonding at the interface are still weaker than those observed in the carbonated aggregate group. The number of ITZ-aligned cracks on the UCS fracture surface is significantly reduced compared to the 14-day sample, yet ITZ still serves as a preferential failure path under compression. While some filling does occur, the overall interfacial continuity and uniform bridging remain inferior to that of the carbonated aggregate group. In contrast, the latter—under the influence of a carbonate shell—tends to form denser, more uniform bridging zones and reduce grayscale contrast at the interface.

Figure 17j shows that the morphology of this fracture surface closely resembles a composite or matrix–aggregate co-fracture mode. The surface is overall more regular; aggregates are not completely pulled out, with blurred contours and visible residual matrix adhering to their surfaces, indicating significantly enhanced interfacial bonding. In some areas, the fracture path crosses directly through the aggregates. Corresponding Micrographs (k) and (l): The ITZ boundary appears densely compacted, with significantly reduced porosity and a clear, continuous interface. The cracks extend vertically along the ITZ. A high-density surface layer is observed near the aggregate boundary, with a density close to or even exceeding that of the matrix, and noticeably higher than that of the non-carbonated group.

After UCS testing, only a few fine cracks are visible, mostly vertically distributed and short-ranged near the interface. These cracks more frequently initiate or terminate near the aggregate’s outer surface, rather than penetrating the interface, suggesting that the interfacial bonding strength has surpassed that of the aggregate’s near-surface region—signaling a shift in failure mode from “interface-controlled” to “composite-controlled.”

The synergistic interaction between gypsum-induced AFt formation and gypsum-facilitated CaCO_3_ shell development via flue gas carbonation significantly densifies and integrates the ITZ [13,50]. Compared to the non-carbonated GA-SC-Conc group, the number and length of cracks are significantly reduced, and the interfacial continuity is clearly improved. These observations confirm that carbonation substantially enhances the structural integrity of the ITZ and its ability to control crack propagation.

### 3.4. Microstructural Evolution Within the Aggregate Region

At 14 days, the unmodified aggregates exhibit a loose microstructure, characterized by angular CFBFA particles, visible inter-particle voids, and sparse hydration products. The prevalence of sharp edges and unfilled pore throats suggests poor gel connectivity and low initial reactivity. Carbonation, however, significantly improves the internal morphology, narrowed inter-pore constrictions, and more uniformly distributed hydration products, indicating early-stage densification of the aggregate. In the gypsum-added but non-carbonated system, abundant needle-like AFt crystals appear in pore structures. When carbonation and gypsum are combined, AFt morphologies become finer, shorter, and better integrated within the C–(A)–S–H gel, contributing to a more cohesive and homogenous aggregate interior at both 14 and 28 days.

By 28 days, continued secondary hydration enhances overall gel content in all systems. Carbonated aggregates show substantial morphological refinement, with the densest and most homogeneous appearance observed in the gypsum–carbonation synergistic system. Here, finely dispersed AFt and C–(A)–S–H networks fill internal cavities effectively, providing microstructural continuity and resistance to crack propagation.

### 3.5. Matrix Microstructure and Ion Transport Dynamics

The matrix at early ages shows limited hydration, with coarse plate-like hydrates and unreacted residues. In gypsum-free systems, the matrix remains immature and loosely packed. However, the addition of gypsum facilitates the formation of abundant needle-like AFt within the matrix, derived from sulfate release by the aggregate. This AFt deposition contributes to local pore filling but remains uneven and loosely organized in the absence of carbonation.

In carbonated systems, the matrix becomes progressively denser, with finer and more evenly distributed AFt crystals embedded within a continuous C–(A)–S–H matrix. The improved uniformity reflects the buffering effect of the carbonate shell on sulfate flux, which mitigates local supersaturation and favors homogeneous nucleation. By 28 days, the co-presence of gypsum and carbonation results in the densest matrix phase among all samples, consistent with improved macro-mechanical performance.

### 3.6. ITZ Morphology, Crack Behavior, and Failure Patterns

At 14 days, the ITZ in unmodified systems displays poor structural definition—marked by coarse surface textures, and unreacted residues. Cracks preferentially propagate along the aggregate–matrix boundary, resulting in a “tearing–pull-out” failure mode, indicative of weak interfacial bonding [51,52]. In the GA-SC-Conc group, although AFt is present, it tends to form coarse clusters with weak lateral confinement, accompanied by microcracks, and fails to form a mechanically integrated transition zone.

Carbonation introduces a CaCO_3_ shell at the aggregate surface, which functions both as a nucleation scaffold and a diffusion buffer for sulfate ions. This modification facilitates the uniform growth of finely dispersed AFt and C–(A)–S–H within the ITZ, reducing porosity and disrupting crack paths. As a result, the failure mode transitions from interface-dominated to matrix-penetrating, with many cracks deviating into the bulk matrix or traversing the aggregate itself. This is particularly evident in the GA-FHC-Conc specimen, where ITZ densification, crack deflection, and the presence of vertical microcracks signify a passivated and mechanically integrated interface.

### 3.7. Synergistic Role of Carbonation and Gypsum on ITZ Optimization

The combined application of gypsum and carbonation yields the most effective ITZ modification. The carbonate shell mitigates rapid ion transport and promotes the spatially uniform deposition of fine hydration products. Concurrently, gypsum supplies sufficient SO_4_^2−^ to sustain AFt formation. This synergy fosters the development of a finely structured interfacial layer composed of short, dense AFt crystals interwoven with C–(A)–S–H gel. The ITZ thus transforms from a loosely bonded transition zone to a compact, cohesive bridge between aggregate and matrix.

This evolution is supported by both microstructural and mechanical indicators. ITZ thickness reduces significantly while the number and length of interfacial cracks are minimized. Crack trajectories shift from linear, interface-aligned propagation to tortuous, matrix-traversing paths, consistent with a transition from “interface-dominated” to “composite-controlled” failure modes.

**Figure 18:**
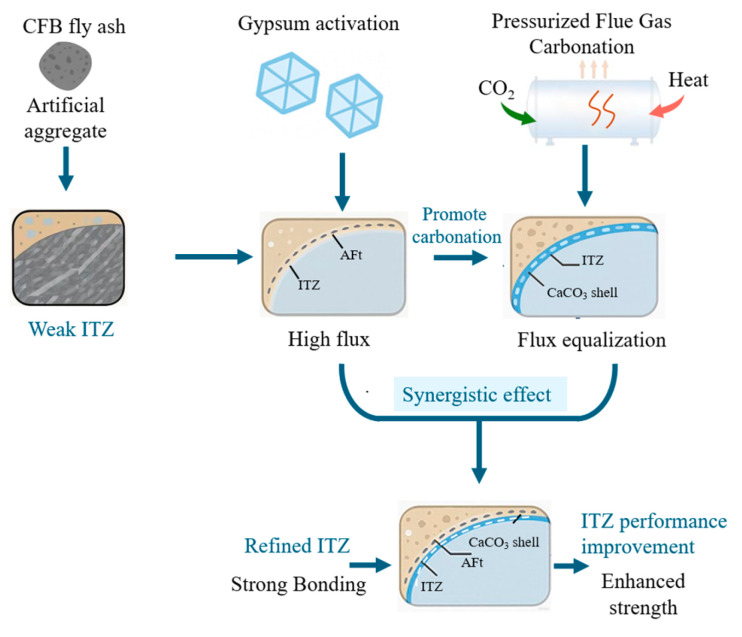
Synergistic mechanism of gypsum activation and pressurized carbonation on ITZ refinement.

Based on the above microstructural observations and mechanistic analyses, the synergistic effect of gypsum activation and pressurized flue gas carbonation on ITZ refinement can be summarized as shown in Figure 18.

## 4. Conclusions

This study presents a robust strategy integrating gypsum activation and flue gas heat curing (FHC) to optimize the interfacial transition zone (ITZ) in high-volume CFBFA-based artificial aggregate concrete.

Methodological Innovation:

A non-contact grayscale-based double-peak gradient method was developed to quantify ITZ thickness, revealing a strong inverse correlation (R^2^ = 0.87) with UCS. This offers a reproducible tool linking microstructure to mechanical performance.

Synergistic Mechanism:

The combined effect of gypsum (AFt formation) and FHC (CaCO_3_ shell formation) densifies the ITZ, enhances aggregate–paste bonding, and shifts failure mode from interface- to composite-controlled fracture.

Sustainability Implications:

This dual-treatment pathway enables the valorization of low-reactivity CFBFA in structural applications, while contributing to CO_2_ sequestration. Future work should address long-term durability and industrial scalability.

Limitations and Directions for Future Research

The proposed FHC–gypsum strategy provides a robust, scalable pathway for engineering sustainable, high-performance cementitious composites using low-reactivity industrial by-products. This strategy not only improves mechanical integrity but also expands the application potential of flue gas carbonation curing in sustainable cementitious composites, offering a scientifically grounded method for interface regulation and long-term durability enhancement. While this study successfully demonstrates the short-term mechanical benefits, a clear limitation is the current focus on 28-day performance. Future research should focus on the long-term durability performance (e.g., carbonation depth and freeze–thaw resistance) and the optimization of the pressure vessel parameters for industrialized application.

## Figures and Tables

**Table 1:** Chemical properties of materials.

Material	CFBFA	Portland Cement	Gypsum	Hydrated Lime
SO_3_	3.075 ± 0.04	4.029 ± 0.02	51.549	0.647
CaO	5.067 ± 0.05	53.678 ± 0.21	43.205	93.886
SiO_2_	46.237 ± 0.25	21.245 ± 0.04	-	0.928
Al_2_O_3_	29.502 ± 0.20	7.298 ± 0.01	0.794	0.956
Fe_2_O_3_	9.875 ± 0.08	4.591 ± 0.01	-	-
MgO	-	6.037 ± 0.02	3.925	2.511

Note: The measured values for CFBFA and Portland Cement are presented as mean Standard Deviation from triplicate XRF measurements. Gypsum and Hydrated Lime are certified analytical-grade reagents whose measured compositions were found to be highly consistent with theoretical stoichiometry, confirming their purity and negligible experimental variability.

**Table 2:** Mix proportions of the reference concrete (per m^3^).

Mix	Dosage
Cement (kg/m^3^)	806
Fine aggregate (kg/m^3^)	396
Coarse aggregate (kg/m^3^)	See Note 1
Water (kg/m^3^)	242
Liquid Superplasticizer (PCE) (L)	19.5

Note 1: The mass of the coarse aggregate was adjusted for each mix to maintain a constant solid volume based on its bulk density, as presented in Table 4.

**Table 3:** Design of CFBFA-based artificial coarse aggregate feedstock ratios under different curing conditions.

Sample	Curing Condition	Cement/wt%	Hydrated Lime/wt%	Gypsum/wt%	CFBFA/wt%
GF-StdC	Standard curing	10	5	0	85
GA-StdC	Standard curing	10	5	5	80
GF-FHC	FHC (1 h) + StdC (28 d)	10	5	0	85
GA-FHC	FHC (1 h) + StdC (28 d)	10	5	5	80

**Table 4:** Physical properties of aggregates.

Sample	Bulk Density(g/cm^3^)	Crushing Strength (MPa)	Water Absorption for 24 h (%)
GF-StdC	1.49	4.37	17.03
GA-StdC	1.74	7.39	6.65
GF-FHC	1.54	5.39	14.26
GA-FHC	1.75	9.13	3.59

## Data Availability

The original contributions presented in this study are included in the article. Further inquiries can be directed to the corresponding authors.
